# Long-Term Socioeconomic Impact of Informal Care Provided to Patients with Pacemakers: Remote vs. Conventional Monitoring

**DOI:** 10.3390/healthcare8020175

**Published:** 2020-06-16

**Authors:** Cesar Leal-Costa, Antonio Lopez-Villegas, Daniel Catalan-Matamoros, Emilio Robles-Musso, Knut Tore Lappegård, Rafael Jesus Bautista-Mesa, Salvador Peiró, Remedios Lopez-Liria

**Affiliations:** 1 Nursing Department, University of Murcia, 30003 Murcia, Spain; cleal@um.es; 2 Social Involvement of Critical and Emergency Medicine, CTS-609 Research Group, Hospital de Poniente, 04700 El Ejido-Almería, Spain; 3 Department of Journalism and Communication, Universidad Carlos III de Madrid, 28903 Madrid, Spain; dacatala@hum.uc3m.es; 4 Health Sciences CTS-451 Research Group, University of Almería, 04120 Almería, Spain; 5 Intensive Care Unit, Hospital de Poniente, 04700 El Ejido-Almería, Spain; emiliomartin.robles-musso@ephpo.es; 6 Institute of Clinical Medicine, Faculty of Health Sciences, University of Tromsø, 9019 Tromsø, Norway; knut.tore.lappegard@gmail.com; 7 Division of Medicine, Nordland Hospital, 8005 Bodø, Norway; 8 Management Unit, Hospital de Poniente, 04700 El Ejido-Almería, Spain; rafaeljesus.bautista@ephpo.es; 9 Health Services Research Unit, FISABIO-PUBLIC HEALTH, 46020 Valencia, Spain; peiro_bor@gva.es; 10 Department of Nursing Science, Physiotherapy and Medicine, Hum-498 Research Team, Health Research Centre, University of Almería, 04120 El Ejido-Almería, Spain; rll040@ual.es

**Keywords:** cost of illness, disease burden, informal caregiving, pacemaker follow-up, remote monitoring, telemedicine

## Abstract

The impact of informal care immediately after pacemaker (PM) implantation has been well established; however, not much is known about its long-term effects. The present study compared personal characteristics, associated problems, workloads, time, and costs related to informal care provided to patients with PM under remote monitoring (RM) vs. conventional monitoring (CM) in the hospital, five years after implantation. The PONIENTE study was a controlled, non-randomized or masked clinical trial conducted with information obtained from the perspective of informal caregivers. Data were collected at 12 and 60 months after PM implantation. The patients in the study were assigned to two different groups: remote monitoring (RM) and conventional monitoring (CM). The “Disability, personal autonomy, and dependency situations survey” (EDAD) was administered to collect information on sociodemographic characteristics, time, care difficulties, health status, professional aspects, and impact on economic, family, or leisure aspects of the main caregivers providing care to patients with pacemakers. After five years, 55 patients completed the study (RM = 21; CM = 34). The average age was 63.14 years (SD = 14.90), 96% of them were women, and the most predominant marital status was married (72%). Informal caregivers lived in the homes of the patients in 70% of cases, and 88% indicated that they had to provide care six to seven days a week. The average cost per patient during the monitoring period studied was 13.17% lower in the RM group than in the CM group, and these differences were not statistically significant (*p* = 0.35). This study found similar results in the two groups under study with respect to sociodemographic characteristics, workload, time, and problems associated with health, leisure and family members. The costs associated with care were higher in the CM group; however, these differences were not statistically significant.

## 1. Introduction

Cardiac pacemaker implantation has increased considerably in recent years [1,2,3,4,5]. The aging of the population has added to this reality, leading patients with pacemakers to exhibit more and more comorbidities and, consequently, a greater need for both formal and informal care [6,7]. Care provided by informal caregivers to individuals with pacemakers is mainly performed by family members. This fact has a significant impact on different aspects such as emotional, physical, and economic [8].

Currently, the technology aimed to facilitate the monitoring of pacemaker recipients is in continuous development. There are remote monitoring (RM) systems that allow the transmission of data stored in the memory of devices [9,10,11,12,13]. However, despite the benefits reported in numerous studies regarding the RM of pacemaker systems [9,10,12], there are still conventional monitoring (CM) programs at hospitals that require patients with pacemakers to periodically go to these institutions to be monitored.

In this way, the demands and the impact affecting informal caregivers can be different if they are providing care to patients under RM or if they are monitoring the patients at hospitals, because the latter requires more effort. The impact of informal care has been previously assessed in a study conducted with a 12-month monitoring period [8]. This way, it is necessary to monitor these patients over longer periods of time to know the problems associated with informal care, real workloads, and the costs five years after pacemaker implantation.

The goal of this study was to assess the differences related to informal care in terms of personal characteristics, associated problems, workloads, time, and costs when providing care to patients with pacemakers five years after implantation, according to the monitoring type performed (monitored remotely or in a conventional manner at hospitals).

## 2. Materials and Methods

### 2.1. Design

The PONIENTE study is a controlled, non-randomized or masked clinical trial conducted between 1 October 2012 and 30 November 2017 in the Hospital de Poniente, El Ejido, Almería, Spain.

### 2.2. Participants

The participants of the study were recruited through convenience sampling. Patients included in the study met the following criteria: (a) aged over 18 years; (b) having signed an informed consent form; (c) having a Medtronic pacemaker implanted, compatible with the Carelink^®^ (Medtronic, Dublin, Ireland) remote monitoring system; and (d) having informal caregivers to meet their needs. On the other hand, the patients excluded were those who: (a) had other types of cardiovascular devices implanted such as implantable automatic defibrillator (ICD); (b) were undergoing cardiac resynchronization therapy (CRT) or using Holter systems; and (c) were participating in other clinical trials.

In this way, 82 patients were selected, of which 76 reported that they had informal caregivers. Finally, during the 60-month monitoring period, 26 participants left, thus leaving a total of 50 caregivers (RM = 21 vs. CM = 29).

### 2.3. Data Collection

One month after pacemaker implantation, all patients had a scheduled visit in the pacemaker consultation where the physician (member of this project) explained to them the characteristics, advantages, and disadvantages of both monitoring modalities and each were offered for their selection. If the patient selected the RM alternative, the cardiologist: (i) programmed the corresponding PM parameters; (ii) explained the use of the Medtronic Carelink^®^ (Medtronic, Dublin, Ireland) monitor and the protocol for sending data to the patient; and (iii) requested the service from the supplier company. According to the PM specifications and physician’s criteria, the patients were asked to submit data at different times. In the RM group, follow-up visits were not scheduled. If the data received detected a cardiac event or a device dysfunction, the patients were contacted via phone and referred to a hospital visit. In the CM group, the patients had visits scheduled according to the cardiologist criterion and the standard practices of the Poniente Hospital.

The patients and their caregivers were interviewed 60 months after pacemaker implantation. Data were collected through personal and/or telephone interviews and were carried out by the same member of the research team and were administered in the same month in which it was five years since the pacemaker was implanted. The instrument used was the survey on disability, personal autonomy, and dependency situations (EDAD, 2008) [14]. This questionnaire was designed by the National Institute of Statistics in Spain, with the purpose of collecting information on the sociodemographic characteristics of the main caregivers. In addition, we obtained information about: (a) characteristics of the care provided; (b) problems associated with informal care such as health deterioration, fatigue, depression, treatment required due to the services provided, or other health problems; (c) professional or economic problems such as loss of jobs, decrease in working hours, problems in meeting work schedules, loss of job opportunities; and (d) problems related to economic, leisure, free time, and family life aspects such as a decrease in leisure time or holidays, personal care, number of friends, or even contact with family members such as children, partners, etc., conflicts with the couple or even not having been able to start a family; (e) workload analysis: caregivers could choose up to five main tasks to describe their main functions; and (f) time and costs of informal care, (i.e., the caregivers estimated the number of hours dedicated to car).

The patients and their caregivers were interviewed 60 months after the pacemaker implantation. Data were collected through personal and/or telephone interviews by members of this research project along the fifth year after PM implantation.

Although there are several methods [15] to estimate the value of time spent on health care provision, in the present study, we used the replacement cost method, which allows calculating the costs involved in replacing informal caregivers with paid home care professionals [16]. To estimate the costs of informal care, the time spent by informal caregivers was linked to data related to gross wages published for 2019 by the Ministry of Employment and Social Security of Spain [17], taking into account the hourly rates of home care. The minimum rate for this activity set for 2019 was €7.04 per hour including Sundays, days off, extra payments, and holidays.

### 2.4. Ethical Considerations

The protocol of the trial and the study were approved by the Regional Ethics Committee for Health Research (CEIC-AL: 53/2012). The present study was conducted in accordance with the precepts of the Declaration of Helsinki [18] and Spanish laws on data protection and patient rights [19,20]. All patients signed the corresponding informed consent form prior to their enrolment, and appropriate measures were taken to ensure the privacy of the data. The trial protocol was registered with ClinicalTrials.gov (Identifier: NCT02234245).

### 2.5. Statistical Analysis

Continuous variables were expressed as means with standard deviations (SDs) and categorical variables were presented as actual numbers and percentages. Caregiver characteristics between groups were compared using a difference in the means test for continuous variables (Mann–Whitney U-test) and a difference in the proportions test (binomial method) or Chi-square test (replaced by Fisher’s exact test for cells with *n* < 5 cases) for qualitative variables. The influence of the variables (age, sex, and type of monitoring) on the dropout rate was assessed using logistic regression. All analyses were performed using SPSS (SPSS Institute, Inc., Chicago, IL, USA) statistical software.

## 3. Results

### 3.1. Main Characteristics of the Informal Caregivers

**Figure 1:**
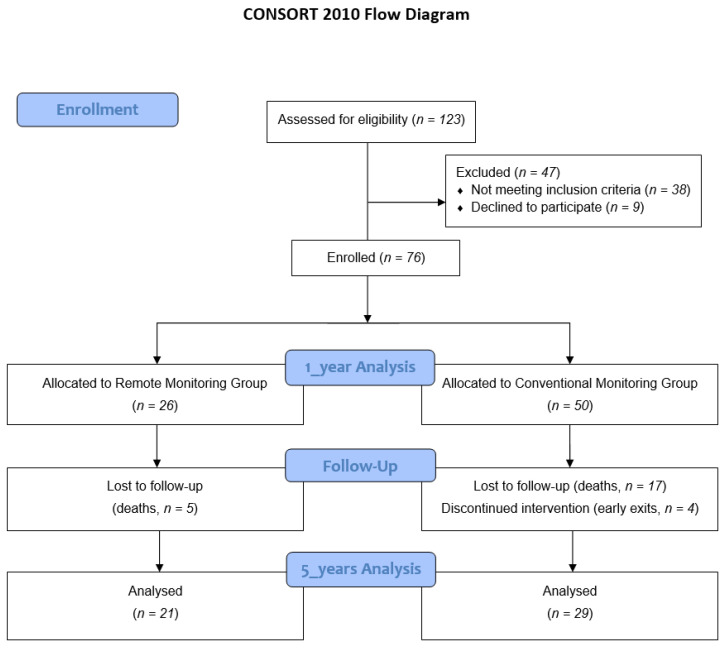
CONSORT flow diagram.

Finally, the sample of caregivers after a 60-month monitoring period consisted of 50 participants (Figure 1), of which 21 (42%) belonged to the RM group and 29 (58%) to the CM group. The average age was 63.14 years (SD = 14.90). With respect to sex ratio, 96% of the participants were women, and their predominant marital status was married (72%). Informal caregivers lived at the homes of the patients and were not domestic workers in 70% of cases. Table 1 shows the main characteristics of informal caregivers.

### 3.2. Attrition

The loss of subjects after 60 months was not related to sex (*p* = 0.91), the age of the participants (*p* = 0.88), or the study criteria variable (i.e., type of monitoring performed (*p* = 0.11)). The results indicated similarity of the sociodemographic characteristics between the participants who left the program and those who remained in it.

### 3.3. Workload and Activities Performed by the Informal Caregivers

**Figure 2:**
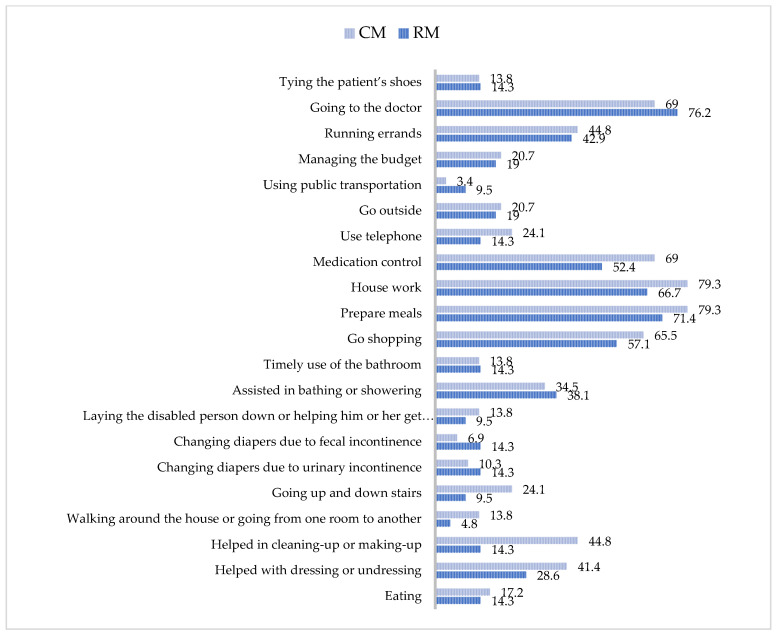
Percentages of activities performed by the informal caregivers. RM = remote monitoring; CM = conventional monitoring.

Based on the answers given by the informal caregivers, the main activities they performed were preparing meals; doing other household chores; going to the doctor; shopping; making arrangements; medication control; helping with bathing/getting ready; and helping with dressing/undressing. The results, according to the monitoring group, indicated that the informal caregivers of the CM group obtained higher percentages than the RM group in tasks such as bathing/getting ready; dressing/undressing; going up or down stairs; lying down/getting out of bed; shopping; doing other household chores; medication control; and using the telephone (Figure 2).

**Figure 3:**
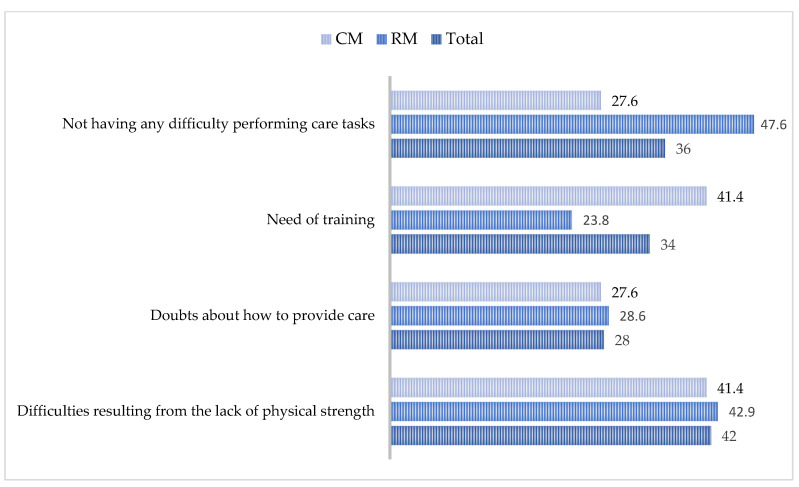
Percentages of the perceptions of informal caregivers about the tasks derived from care. RM = remote monitoring; CM = conventional monitoring.

With respect to the tasks derived from care, 42% of the caregivers reported having difficulties resulting from the lack of physical strength, 28% had doubts about how to provide care, and 34% believed they needed more training. Only 36% of them affirmed that they had no difficulties in performing care tasks. These results indicate that the percentages were similar in the two monitoring groups, except for: (a) the need for training, given that the RM and CM groups obtained 23.8 and 41.4%, respectively, with no significant differences (*p* = 0.16); and (b) not having any difficulty performing care tasks, in which case the RM and CM groups obtained 47.6 and 27.6%, respectively, (*p* = 0.12) (Figure 3).

Many caregivers reported having no problems associated with informal care provided to patients with pacemakers. With respect to health problems or general status, 46% reported general health problems, 22% acknowledged that they had undergone deterioration of health status, and 16% reported tiredness problems. Regarding problems related to leisure, free time, and family life, 46.9% of the informal caregivers reported a decrease in leisure time, 26.5% reported a decrease in holiday time, and 16.3% reported a lack of time for providing care to other individuals (children, husbands, etc.). Finally, regarding professional and economic problems, 14.3% of the caregivers affirmed that they could not work outside the home due to the care tasks, 12.5% had to reduce their working hours, and 8.2% had problems with working hours and lost job opportunities. Only 4.1% had to leave their jobs.

Depending on the monitoring group, it was observed that the CM group achieved a higher percentage in problems related to professional, economic, leisure, free time, and family life aspects, in comparison to the RM group (Table 1).

### 3.4. Time and Costs of Informal Care

Among the informal caregivers of the present study, 88% indicated that they had to provide care six to seven days a week. The caregivers of the RM group worked less hours per day than the CM group (M = 15.14 [SD = 8.38] vs. M = 17.03 [SD = 6.73], respectively) with no statistically significant differences. Regarding the number of years during which the participants had been providing care, it was observed that only 38% of them had performed that activity between four and eight years or more. This fact would indicate a change in the roles of the main caregivers after five years. This way, 62.2% of the caregivers would have been taking care for less than one to four years; however, there was not an association between the variables ‘type of monitoring’ and ‘time in years’ during which the participants had been providing care (Table 2).

Finally, the average cost per patient during the monitoring period studied was lower for patients of the RM group (RM = €170,086.40 [SD = 108,009.37]; [95% CI = €89,862.15–€198,729.29]) than for the patients of the CM group (CM = 195,877.08 [SD = €15,504.03]; [95% CI = €115,652.83–€224,519.97]). It is worth noting that these differences were not statistically significant (Table 2).

## 4. Discussion

In the present study, the possible differences related to informal care were assessed 60 months after the implantation of the pacemakers by assessing the participants of the two groups (i.e., RM and CM), highlighting the personal characteristics, associated problems, workloads, time, and economic cost of informal care provided to patients with pacemakers.

Sample loss occurred as in the majority of studies with long monitoring periods. In the present case, 26 participants left the program, although the binary logistic regression analysis showed results that suggested similarity in the variables ‘sociodemographic characteristics’, ‘sex’, and ‘age’ of the two groups, and in the variable ‘monitoring type’ between participants who dropped out and those who remained in the monitoring program.

The profile of the informal caregivers after the 60-month monitoring period indicated that most of them were women, married, with an average age of 63 years, living in the patients’ homes, and were not domestic workers (70%). These characteristics are similar to those reported in other studies conducted with informal caregivers of individuals with heart failure [21], patients with pacemakers [8], or patients with other chronic illnesses [14,22].

The caregivers of our study reported health problems associated with informal care as well as professional or economic problems in terms of leisure, free time, and family life. Among the participants, 46% reported health problems, 22% reported deteriorated health, and 16% reported physical fatigue. These data are consistent with those obtained in other studies conducted with informal caregivers of chronic patients with heart [21,23,24], respiratory [24,25], or other chronic illnesses [14,26].

The caregivers included in the CM group had a higher percentage of health-related problems, although there was no statistically significant association. In a previous study conducted with informal caregivers of patients with pacemakers monitored for a year [8], the caregivers of the CM group reported having more problems associated with health. These data are similar to those obtained in the present study, even though the percentage was lower. This result may be due to the fact that, over time, caregivers adapt to the care tasks required by patients with pacemakers.

With respect to problems related to leisure, free time, and family life, a high percentage of informal caregivers reported the lack of time for leisure (46.9%), for holidays (26.5%), and for providing care to other individuals (16.3%). Regarding professional or economic problems, few participants affirmed that they had them, the most relevant being those related to the inability to work outside the homes, (14.3%) and having to reduce their working hours (12.5%). These results indicate that the social and professional well-being of caregivers can be affected by the tasks derived from informal care, in a manner consistent with the results of other similar studies conducted with chronic patients [14,25,27,28,29].

The workload of informal caregivers was related to the tasks they performed to help meet the basic and instrumental needs of the daily life of patients with pacemakers, namely: preparing meals; doing household chores; going to the doctor; shopping; managing procedures; medication control; helping with bathing/getting ready; and helping with dressing/undressing—it is worth noting that these tasks have been described by other informal caregivers in simulated studies [14,21,29,30,31]. There was a higher percentage of caregivers who performed these tasks in the CM group, and these results were similar to those obtained in previous studies conducted with a one-year monitoring period [8,32].

On the other hand, at the time of providing care, 28% of the participants had doubts about how to perform the tasks, and 34% expressed the need to obtain more training. These results are consistent with those of other studies [21,30,31], in which informal caregivers experienced ambiguity and uncertainty with respect to the caregiver’s role. In addition, they were afraid of making serious mistakes due to their lack of competence to perform care tasks correctly.

The average time dedicated to provide care was 16.24 h per day (SD = 7.45) across the whole sample. The distribution according to the monitoring groups was 15.14 h (SD = 8.38) in the RM group and 17.03 h (SD = 6.73) in the CM group, without statistically significant differences between the two groups. These results are similar to the findings of other studies that have quantified the daily hours dedicated to informal care provided to patients with pacemakers such as the study conducted by López-Villegas et al. [8], who reported an average of 15.61 h per day in the whole sample. In the present study, the caregivers of the CM group spent more hours than the caregivers of the other monitoring group. On the other hand, Ricci et al. [32] described a total loss of working hours or activities of informal caregivers, which had been greater in the CM group. These differences between the RM and CM groups were statistically significant in a 12-month monitoring period.

Regarding the costs associated with informal care, they followed a pattern similar to that of the daily hours dedicated to informal care. The CM group had a higher cost after the 5-year monitoring period in comparison to the RM group. In this way, we observed an increase of 15.16% in the costs of the CM group. The results obtained in the present study indicated a smaller impact in comparison to those impacts found in previous studies in which monitoring had been performed for twelve months [8,32] in patients with pacemakers, and in studies that had monitored patients with cardiac insufficiency for six and twelve months [33,34]. Although the cost differences found were smaller in the RM group, no statistically significant differences were found with respect to the CM group. On the other hand, significant differences were found in previous studies [8,32] conducted with patients with pacemakers during a shorter monitoring period (12 months). In the light of the results found, it is possible to affirm that, in the RM group, cardiovascular events were detected before and there was a reduction in the number of hospitalizations, hospital visits, and possible costs associated with the monitoring of these patients with pacemakers. This fact was reflected in the overload of informal caregivers. On the other hand, these results are similar to those obtained in previous studies that had assessed the economic impact of RM provided to patients with pacemakers [5,8,9,32,34,35,36].

### Limitations

The PONIENTE study has some limitations. First, the selection of participants was not chosen at random, given that the decision on the type of monitoring was decided by consensus between the patients and the physicians. Although no significant differences were found between the two groups at the beginning of the study, certain variables not observed such as cultural level, rural location/distance to hospital, level of patient dependence, etc., could have affected the results (indication of bias). However, the method of non-randomization used in the present study, based on daily practice, provided results that may be closer to those achieved in the real world than in comparison to those obtained through randomization (greater external validity vs. lower internal validity) [8].

Second, the number of participants enrolled in this single-center trial was limited by the number of implants per year and the number of patients who had informal caregivers, in addition to the loss of sample size after the 5-year monitoring period. For this reason, we assessed attrition, demonstrating the similarity in the sociodemographic characteristics and the type of monitoring between the participants who left and those who remained in the program.

Third, it was not possible to confirm the causal effect between the implantation of pacemakers and the overload of informal care, given that there could have been mediating and moderating effects of other variables that could influence the results obtained. For this reason, the results should be taken into account with caution, and other studies should be conducted with larger and multi-center samples in which these possible mediation and moderation effects could be assessed.

Despite the limitations described, this is the first study that assessed the impact of informal care in patients with pacemakers in the health, social, professional, and economic dimensions after a 5-year monitoring period, making a differentiation between the participants monitored remotely or conventionally at the hospitals.

## 5. Conclusions

Five years after the implantation of the pacemakers, the informal caregivers included in the two monitoring groups achieved similar results with respect to sociodemographic characteristics, problems associated with healthcare, work, leisure, family, workload, and working time. However, the results were generally greater in the CM group.

The costs associated with informal care provided to patients with pacemakers and monitored remotely were lower than those obtained by the conventional monitoring group. However, these differences were not statistically significant.

## Figures and Tables

**Table 1:** Characteristics and problems associated with informal care provided to patients with pacemakers, according to the total number of participants and those in the monitoring groups.

Variables	Total *n* = 50		Remote monitoring *n* = 21		Conventional monitoring *n* = 29		*p*
Age (mean, SD)	63.14	14.9	62.9	15.14	63.32	14.99	0.92
Sex (*n %*)							
Male	2	4	2	9.5	0	0	0.17
Female	48	96	19	90.5	29	100	
Marital status (*n*, *%*)							
Single	10	20	5	23.8	5	17.2	0.77
Married	36	72	14	66.7	22	75.9	
Widower	4	8	2	9.5	2	6.9	
Separated	0	0	0	0	0	0	
Divorced	0	0	0	0	0	0	
Type of caregiver (*n*, *%*)							
Lives at the patient’s home-non-domestic worker	35	70	17	81	18	62.1	0.49
Lives at the patient’s home-domestic worker	1	2	0	0	1	3.4	
Does not live at the patient’s home-non-domestic worker	11	22	3	14.3	8	27.6	
Does not live at the patient’s home-domestic worker	3	6	1	4.8	2	6.9	
Health problems-general status (*n*, *%*)							
Deterioration of health status	11	22	5	23.8	6	20.7	0.53
Fatigue	8	16	4	19	4	13.8	0.45
Depression	2	4	1	4.8	1	3.4	0.67
Treatment	2	4	2	9.5	0	0	0.17
Other health problems	23	46	10	47.6	13	44.8	0.54
Professional or economic problem (*n*, *%*)							
Loss of employment	2	4.1	0	0	2	7.1	0.32
Reduction of working hours	6	12.5	1	4.8	5	18.5	0.16
Problems with working time	4	8.2	1	4.8	3	10.7	0.42
Cannot work out of the home	7	14.3	2	9.5	5	17.9	0.35
Economic problems	3	6.1	1	4.8	2	7.1	0.61
Loss of employment opportunities	4	8.2	1	4.8	3	10.7	0.42
Problems related to leisure, free time, or family life (*n*, *%*)							
Reduction in leisure time	23	46.9	7	33.3	16	57.1	0.09
Reduction in holidays time	13	26.5	4	19	9	32.1	0.24
Time for providing care to other individuals	8	16.3	1	4.8	7	25	0.62
Time with friends	4	8.2	1	4.8	3	10.7	0.42
Time for self-care	2	4.1	1	4.8	1	3.6	0.68
Conflicts with the couple	1	2	1	4.8	0	0	0.43

**Table 2:** Costs of informal care provided to patients with pacemakers.

Variables	Total *n* = 50		Remote Monitoring *n* = 21		Conventional Monitoring *n* = 29		*p*
Hours per day (mean, SD)	16.24	7.45	15.14	8.38	17.03	6.73	0.38
Days per week (*n*, *%*)							
<1 day	1	2	1	4.8	0	0	0.55
1 day	1	2	1	4.8	0	0	
2–3 days	2	4	1	4.8	1	3.4	
4–5 days	2	4	1	4.8	1	3.4	
6–7 days	44	88	17	81	27	93.1	
Hours per week (mean, SD)	109.52	55.87	100.67	63.93	115.93	49.41	0.35
Years (*n %*)							0.85
<1	7	14	4	19	3	10.3	
From 1 to 2	10	20	5	23.8	5	17.2	
From 2 to 4	14	28	5	23.8	9	31	
From 4 to 8	16	32	6	28.6	10	35.5	
>8	3	6	1	4.8	2	6.9	
Informal costs (€) (mean, SD)	185,044.99	94,394.59	170,086.40	108,009.37	195,877.08	15,504.03	0.35
